# Surfactant-Templated Synthesis of Polypyrrole Nanocages as Redox Mediators for Efficient Energy Storage

**DOI:** 10.1038/srep14097

**Published:** 2015-09-16

**Authors:** Ki-Jin Ahn, Younghee Lee, Hojin Choi, Min-Sik Kim, Kyungun Im, Seonmyeong Noh, Hyeonseok Yoon

**Affiliations:** 1 Alan G. MacDiarmid Energy Research Institute, School of Polymer Science and Engineering, Chonnam National University, 77 Yongbong-ro, Buk-gu, Gwangju 500-757, South Korea; 2 Department of Polymer Engineering, Graduate School, Chonnam National University, 77 Yongbong-ro, Buk-gu, Gwangju 500-757, South Korea

## Abstract

Preparation of conducting-polymer hollow nanoparticles with different diameters was accomplished by surfactant templating. An anionic surfactant, namely sodium dodecylbenzenesulfonate, formed vesicles to template with the pyrrole monomer. Subsequent chemical oxidative polymerization of the monomer yielded spherical polypyrrole (PPy) nanoparticles with hollow interiors. The diameter of the hollow nanoparticles was easily controlled by adjusting the concentration of the surfactant. Subsequently, the size-dependent electrochemical properties of the nanoparticles, including redox properties and charge/discharge behavior, were examined. By virtue of the structural advantages, the specific capacitance (max. 326 F g^−1^) of PPy hollow nanoparticles was approximately twice as large as that of solid PPy nanospheres. The hollow PPy nanostructure can easily be used as a conductive substrate for the preparation of metal/polymer nanohybrids through chemical and electrochemical deposition. Two different pseudocapacitive metal-oxide clusters were readily deposited on the inner and outer surfaces of the hollow nanoparticles, which resulted in an increase in the specific capacitance to 390 F g^−1^. In addition, the hollow nanoparticles acted as a nanocage to prevent metal ion leaching during charge/discharge, thus allowing an excellent capacitance retention of *ca.* 86%, even following 10,000 cycles.

Nanostructures are of interest in a wide range of applications, ranging from optoelectronic to electrochemical devices. In the past few decades, considerable attention has been focused on energy storage devices based on electrochemical mechanisms, such as secondary batteries and supercapacitors, because of their potential application in hybrid electric vehicles (HEVs) or portable electronic devices^1^,^2^,^3^,^4^,^5^. Specifically, nanostructured materials have attracted particular interest due to their high energy density, excellent rate capability, and long cycle life^4^,^5^,^6^,^7^,^8^,^9^,^10^. Hollow nanoparticles can be utilized for a variety of applications in areas such as encapsulation, catalysis, coating, and composite science^11^,^12^,^13^,^14^,^15^,^16^. The huge demand for hollow nanoparticles has expedited the development of various synthetic strategies, which can be categorized into two main classes, namely hard template and soft template syntheses. The most common technique has been the hard template route, employing templates such as silica, gold, and polymeric nanoparticles^17^,^18^,^19^,^20^,^21^,^22^,^23^. The majority of these approaches involve multistep synthetic routes to pre-modify core templates, and to remove the core by either heating, or chemical treatment. The overall process is therefore rather tedious, and is not suitable for large-scale production. In addition, the subsequent removal of the template may result in the deformation of the resulting product. Compared to the hard template approach, the soft template approach is relatively straightforward, although it is somewhat difficult to precisely control the size and shape of the resulting product. Soft template approaches include micro-/mini-emulsion polymerization, layer-by-layer self-assembly, phase separation of block copolymers, cross-linking of micellar structures, and vesicle polymerization^24^,^25^,^26^.

Currently, there is huge demand for the development of materials and devices for enhanced energy storage, and a great amount of research effort has been devoted to achieving high performance electrode materials and devices^27^,^28^. From a materials science point of view, electroactive conducting polymers are considered a promising organic material for such devices because of their favorable electrical conductivity, physicochemical properties, and environmental durability. Conducting polymers that can store electric charge by a redox pseudo-capacitive mechanism have been explored for use in rechargeable energy storage devices due to their exclusive advantages, such as facile synthesis, structural diversity, low weight, and flexibility^29^,^30^,^31^. Recent advances in nanotechnology have allowed for the widespread use of nanoparticles in electrodes for energy storage and conversion applications^32^,^33^. In particular, nanostructuring of conducting polymers can increase the contact area between the polymer and the electrolyte, resulting in enhanced kinetic characteristics and charge storage capacity. In general, favorable electron pathways are extremely important for efficient charge transport, and therefore to obtain a high specific capacitance from a pseudocapacitive material. Polypyrrole (PPy), for example, is regarded as one of the most promising conducting polymers for industrial applications. However, like many other conducting polymers, PPy is both infusible and insoluble in common solvents. Therefore, control on the morphology of conducting polymers during polymerization is particularly important and remains a challenge. To date, a number of approaches have been made to prepare conducting polymer nanostructures with desirable morphology and properties^34^,^35^,^36^,^37^,^38^,^39^,^40^,^41^,^42^. Nevertheless, limited information exists on the preparation of hollow nanostructures consisting of conducting polymers.

Herein, we report the syntheses of spherical PPy nanoparticles with hollow interiors using surfactant templating, in which an anionic surfactant will be used to build up vesicles as a template for the hollow nanostructures. The diameter of the hollow nanoparticles will be controlled by adjusting the concentration of the surfactant, and their size-dependent electrochemical properties will be examined in terms of redox properties and charge/discharge behavior. Compared to solid nanostructures, the hollow polymer nanostructure is advantageous for energy storage applications by virtue of the effective inner/outer surfaces and inner void. In order to take further advantage of the hollow nanostructure, the deposition of metal nanoclusters on the nanoparticles will be carried out using two different routes, namely electrodeposition and chemical deposition. We expect that the resulting metal/polymer hollow nanoparticles will be demonstrated to be promising electrode materials for high-performance electrochemical capacitors. It should also be noted that at present, little research has been carried out on the use of hollow conducting polymer nanostructures for energy storage applications, mainly due to difficulties in their preparation.

## Results and Discussion

**Figure 1:**
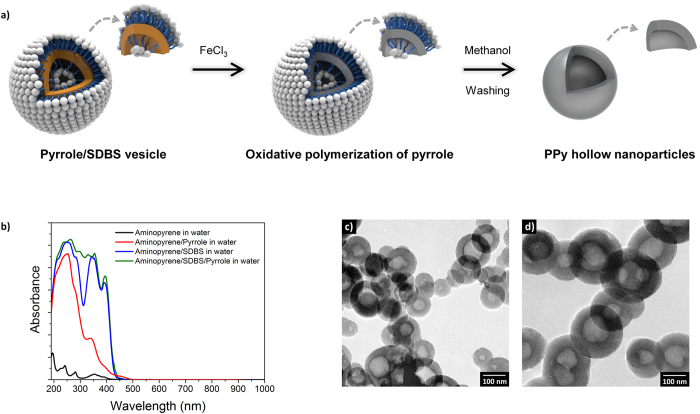
Preparation of PPy hollow nanoparticles. (a) Schematic demonstration of the mechanism of formation; (b) UV-Vis absorption spectra of aminopyrene in different microenvironments; (c) TEM image of the hollow nanoparticles with 100 nm diameter; and (d) 200 nm diameter.

Spherical PPy hollow nanoparticles were readily obtained *via* surfactant templating in aqueous solution. It was found that increasing the molar ratio of pyrrole to sodium dodecylbenzenesulfonate (SDBS) resulted in an increase in the diameter of the hollow nanoparticles. Figure 1a depicts a plausible mechanism of formation of the hollow nanoparticles in the surfactant solution. In the initial stage, the pyrrole monomer is dispersed and emulsified with the anionic surfactant (SDBS) in aqueous solution. To obtain the nanospheres, the surfactant molecules should be assembled into spherical micelles or vesicles. Considering the dimensions of the resulting nanospheres, it appears that spherical micelles composed of a monolayer palisade of surfactant molecules are not suitable as a template. This is likely due to the structural characteristics of SDBS, which make vesicle formation more favorable. Since the diameter of the hollow nanoparticles depended on the concentration of pyrrole relative to the surfactant, pyrrole would also contribute to the formation of SDBS vesicles. Pyrrole, a five-membered nitrogen heterocyclic ring, has a low water-solubility (60 g L^−1^) but a somewhat high dipole moment (1.8 D). Thus, pyrrole can theoretically be located between the hydrophobic head-groups of the surfactant molecules through secondary forces such as π−π interactions and hydrogen bonding, which leads to a decrease in the effective area of the head-group. As a result, the increased packing parameter for the individual surfactant molecules makes them favorable to the formation of larger vesicles.

The fluorescent probe aminopyrene was then employed to obtain information relating to vesicle formation and the relative position of the monomer in solution. Aminopyrene was particularly suitable for this role as it has a high chemical affinity with pyrrole. Figure 1b shows the fluorescence spectra of aminopyrene solubilized in three different microenvironments. When solubilized in water, aminopyrene gave seven fluorescence absorption peaks (224, 242, 270, 282, 352, 370, and 390 nm). Upon the addition of SDBS or/and pyrrole, the fluorescence underwent perturbations in peak intensity and position. Remarkably, the intensity ratios of the 270 nm peak to the 282 nm peak, and that of the 390 nm peak to the 370 nm peak increased upon the sequential addition of SDBS and pyrrole. From these changes in fluorescence, it appears that aminopyrene is indeed positioned in three different microenvironments, implying that the vesicle templates are generated with the aid of both SDBS and pyrrole. Technically, it is not facile to obtain polymeric hollow nanospheres *via in situ* polymerization on the soft surfactant template. However, the polymerization rate of PPy is rapid due to the low oxidation potential of the monomer, leading to the generation of the hollow nanostructure prior to collapse of the template during polymerization. Consequently, PPy hollow nanospheres with diameters of 100 ± 15 and 200 ± 26 nm were prepared employing varying molar ratios of pyrrole to SDBS in aqueous solution. As shown in Fig. 1c,d, the TEM images visualize the hollow interiors of the nanospheres. Interestingly, several nanospheres contained multi-segmented interiors (see Supporting Information) due to the irreversible aggregation of smaller vesicles.

**Figure 2:**
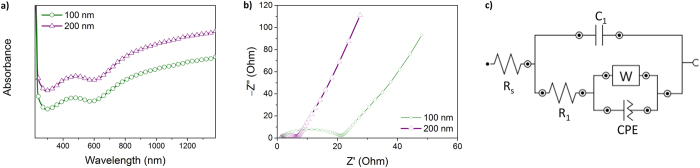
(**a**) UV-Vis-NIR absorption spectra. (**b**) EIS Nyquist plots of the PPy hollow nanoparticles. (**c**) Equivalent circuit model used for fitting the data from (**b**). For the EIS measurements, the nanoparticles were coated on a stainless steel electrode and 1 M H_2_SO_4_ solution was used as an electrolyte.

The intrinsic characteristics of conducting polymer nanoparticles, such as doping and oxidation level, have been shown to determine their electrical and electrochemical properties^8^,^9^. In general, the nanoparticles have to first be assembled prior to integration into the desired devices, in which the size and morphology of the nanoparticles greatly affect the performance of said devices. Consequently, extrinsic factors such as the size and morphology of the nanoparticles also affect the performance of the nanoparticle-based devices. To examine both the intrinsic and extrinsic factors of our nanoparticles, the hollow nanoparticles were characterized by Ultraviolet-visible (UV-Vis) near infrared (NIR) spectroscopy and electrochemical impedance spectroscopy (EIS), as shown in Fig. 2. The UV-Vis-NIR spectra, which provide information on the oxidation level of the nanoparticles, can be seen in Fig. 2a. It was clear that the two nanosphere samples examined showed similar absorption profiles. From the spectra, we can deduce that the absorption at 470 nm is due to the π−π* transitions and the intense absorption in the NIR region can be attributed to polaronic and bipolaronic transitions. As a result, it could be deduced that the oxidation levels of individual nanospheres were comparable and they were present in a highly oxidized state. EIS measurements were then carried out to gain a better understanding of the electrochemical properties of the electrodes consisting of PPy hollow nanoparticles (Fig. 2b). The hollow nanoparticles produced Nyquist plots composed of a semicircle at high frequencies followed by a diagonal line at low frequencies, which is the typical form of a Randles circuit for conducting polymers in an electrolyte. However, the size of the semicircular plot at the real impedance component (Z’) was different for each hollow nanoparticle. The charge transfer resistance (R_ct_) is known to be affected by the polymer itself, the polymer/electrolyte interface, and even by the presence of electrolyte in the interparticle pores. It was found that the R_ct_ of the 100 nm diameter hollow nanoparticles (100-HNPs) was approximately three times higher than that of the 200 nm diameter hollow nanoparticles (200-HNPs). An equivalent circuit model (Fig. 2c) was then used to further interpret the EIS data. It was found that the resistance R_1_ (normally corresponds to R_ct_) of the 200-HNPs in the circuit model was 3.7 times lower than that of the 100-HNPs. The 100-HNPs theoretically have a higher surface area than the 200-HNPs. Nevertheless, the capacitance (C_1_) of the 200-HNPs was 1.3 times higher than that of the 100-HNPs. As a result, we could conclude that the hollow nanoparticles displayed different electrochemical properties when they were aggregated in bulk electrodes, and this behavior may be attributed to interparticle resistance and pore structure.

**Figure 3:**
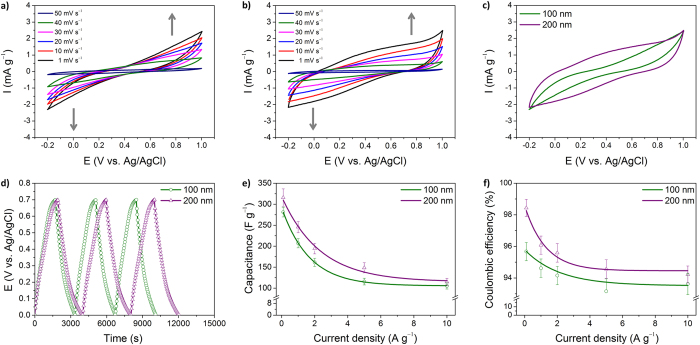
Electrochemical properties of PPy hollow nanoparticles in 1 M Na_2_SO_4_ electrolyte. CV curves of (**a**) 100-HNPs and (**b**) 200-HNPs. (**c**) Representative CV curves recorded at a scan rate of 50 mV s^−1^. (**d**) Representative galvanostatic charge/discharge curves measured at a current density of 0.1 A g^−1^ in a three-electrode cell. (**e**) Specific capacitances measured at different current densities. (**f**) The calculated coulombic efficiencies for both nanoparticles.

To provide further in-depth information on the electrochemical properties, cyclic voltammetry (CV) analysis was carried out in a 1 M Na_2_SO_4_ electrolyte. Figure 3a,b show the CV curves measured at a range of scan rates from 1–50 mV s^−1^. It was found that all curves had similar shapes, with a pair of broad redox peaks being observed for each sample. For a representative comparison, the CV curves recorded at a scan rate of 50 mV s^−1^ are shown in Fig. 3c. The integrated area of the CV curves, indicative of the capacitance of the electrode, was larger for the 200-HNPs than for the 100-HNPs. Galvanostatic charge-discharge measurements were carried out to directly calculate the specific capacitance of the hollow nanoparticles from the discharge curve. Figure 3d shows the typical galvanostatic charge/discharge profiles of the hollow nanoparticle electrodes recorded at a current density of 0.1 A g^−1^, thus allowing a direct comparison between the capacitive behavior of individual samples. The specific capacitances were determined to be 208 and 326 F g^−1^ at 0.1 A g^−1^ for 100-HNPs and 200-HNPs, respectively. Subsequently, the charge/discharge behavior was further explored between 0.1 and 10 A g^−1^. The calculated specific capacitance and coulombic efficiency can be seen plotted against current density in Fig. 3e,f, respectively. It was observed that the specific capacitance decreased gradually at higher current densities. However, the specific capacitance of the 200-HNPs was still higher than that of the 100-HNPs over the entire current density range. In addition, the coulombic efficiency of the 200-HNPs was found to be higher than that of the 100-HNPs. Consequently, we could conclude that the 200-HNPs displayed improved capacitive performance over the 100-HNPs, which was in agreement with the EIS result. In general, the electrode performance can therefore depend on the properties of individual nanoparticles and the assembled status of the nanoparticles on the electrode. As previously mentioned during the discussion of the UV-Vis-NIR spectroscopy data, the oxidation levels of the two different hollow nanoparticles were comparable. Therefore, compared to the 200-HNPs, the capacitive performance of the 100-HNPs would be reduced due to the higher interparticle resistance and slower ion diffusion/exchange rate in the electrode.

**Figure 4:**
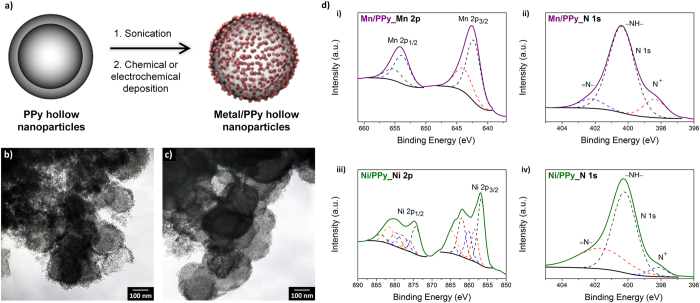
Metal-deposited PPy hollow nanoparticles. (a) Schematic illustration of metal decoration inside and outside the hollow nanoparticles; (b) TEM image of 200-HNPs with chemically deposited manganese nanoclusters; (c) Electrodeposited nickel nanoclusters; and (d) Representative high-resolution XPS spectra.

The rational introduction of functional components into conducting polymers has been known to enhance the inherent properties of the conducting polymers^6^. Here, to take advantage of the hollow structure, manganese and nickel species were deposited in the inner and outer shells of the 200-HNPs through chemical and electrochemical routes, respectively, as shown in Fig. 4a. Initially, sonication of the hollow nanoparticles with an aqueous solution of KMnO_4_ readily generated manganese nanoclusters on the PPy, as can be seen from the transmission electron microscopy (TEM) image in Fig. 4b. As conducting polymers such as PPy have reversible redox properties, this has allowed the polymers to be decorated with metals through use of metal precursors that can act as oxidizing agents. When a strong oxidizing agent such as KMnO_4_ meets PPy, PPy is further oxidized by KMnO_4_, resulting in the formation of manganese nanoclusters on the PPy by simultaneous reduction of KMnO_4_^15^,^27^. Figure 4c shows the TEM image of nickel-decorated PPy hollow nanoparticles. These were formed by dispersion of a nickel precursor, namely Ni(NO_3_)_2_, with the hollow nanoparticles by sonication in an acidic solution, followed by the application of a negative potential (*vs.* Ag/AgCl) to the solution, leading to the formation of nickel nanoclusters at the PPy. X-ray photoelectron spectroscopy (XPS) analysis was carried out to qualitatively characterize the metal/PPy hollow nanoparticles (Fig. 4d)^43^,^44^,^45^. The main spin-orbit components (2p_1/2_ and 2p_3/2_) of manganese were observed at 654.3 and 642.6 eV on the XPS Mn 2p spectrum of the manganese species-decorated PPy (Mn/PPy) hollow nanoparticles. The splitting between the two components was measured as 11.7 eV, suggesting that the predominant oxidation state of the decorated manganese was Mn(IV), in the form of MnO_2_. The high-resolution Ni 2p_3/2_ XPS spectrum of the nickel species-decorated PPy (Ni/PPy) hollow nanoparticles revealed that four different nickel species were electrodeposited. The Ni 2p_3/2_ component of the nickel-decorated PPy hollow nanoparticles can be determined based on peaks at 856.7, 858.5, 860.2, and 862.0–864.7 eV, corresponding to Ni(OH)_2_, nickel metal, NiO, and Ni_2_O_3_, respectively. Considering the contribution based on the peak areas, the predominant oxidation states of the decorated nickel were Ni(II) and Ni(III). In addition, the N 1s XPS spectra of Mn/PPy and Ni/PPy hollow nanoparticles were used to gain information on the oxidation levels of the PPys. Each spectrum displayed three discrete peaks at approximately 398, 400, and 402 eV, attributable to the quinonoid imine (=N−), benzenoid amine (−NH−), and positively charged nitrogen (N^+^), respectively. The atomic ratios of N^+^ to total N species calculated for Mn/PPy and Ni/PPy hollow nanoparticles were 0.17 and 0.09, respectively, which means that the PPy oxidation level of Mn/PPy hollow nanoparticles was higher than that of Ni/PPy hollow nanoparticles. Generally, the oxidation level of conducting polymers is known to be proportional to their electrical/electrochemical properties. As a result, it was demonstrated that functional metal nanoclusters could be introduced to the PPy hollow nanoparticles without significant degradation of the properties.

**Figure 5:**
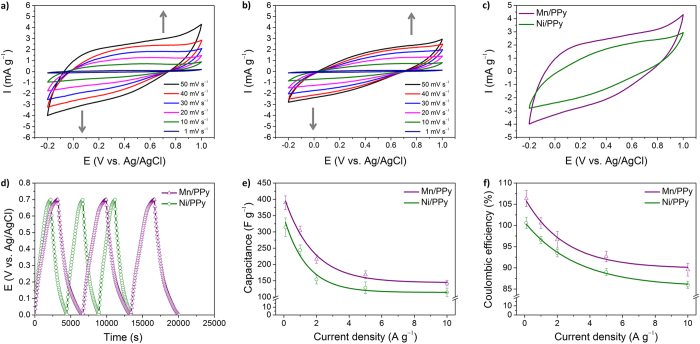
Electrochemical properties of metal/PPy hollow nanoparticles in 1 M Na_2_SO_4_ electrolyte. CV curves of (**a**) Mn/PPy and (**b**) Ni/PPy hollow nanoparticles, and (**c**) representative CV curves recorded at a scan rate of 50 mV s^−1^. (**d**) Representative galvanostatic charge/discharge curves measured at a current density of 0.1 A g^−1^ in a three-electrode cell. (**e**) Specific capacitances measured at different current densities, and (**f**) the calculated coulombic efficiencies.

The metal-decorated PPy hollow nanoparticles could be applied for use in electrode materials for pseudocapacitors. We therefore chose to carry out CV analysis in a 1 M Na_2_SO_4_ solution (the electrolyte) to examine the electroactivity of the metal/PPy hollow nanoparticles. The effect of the potential scan rate on the peak current was monitored at 1–50 mV s^−1^, as can be seen in Fig. 5a,b. Figure 5c displays two representative CV curves of Mn/PPy and Ni/PPy hollow nanoparticles at the same scan rate. It was found that both metal/PPy hollow nanoparticles showed good reversible redox activities. However, the Mn/PPy hollow nanoparticles gave an enlarged CV curve compared with the Ni/PPy ones. The specific capacitance of the metal/hollow nanoparticles was then determined from galvanostatic charge/discharge measurements. For comparison, typical galvanostatic charge/discharge curves of the metal/PPy hollow nanoparticles recorded at a current density of 0.1 A g^−1^ are shown in Fig. 5d. It was found that the discharge time for the Mn/PPy hollow nanoparticles was longer than that for the Ni/PPy hollow nanoparticles, from which curves the specific capacitances were calculated to be 390 and 314 F g^−1^ for Mn/PPy and Ni/PPy hollow nanoparticles, respectively. The charge/discharge curves were further measured between 0.1 and 10 A g^−1^, and the discharge specific capacitance and coulomb efficiencies calculated from these curves can be seen in Fig. 5e,f. From these results, it was clear that metal decoration made it possible to enhance the specific capacitance of the PPy hollow nanoparticles by a maximum of 40% (Mn/PPy 25–40%, Ni/PPy 1–26%). The coulombic efficiency was also found to increase by 1–8% following metal decoration. In particular, we observed that Mn/PPy hollow nanoparticles displayed a better capacitive performance than Ni/PPy ones.

**Figure 6:**
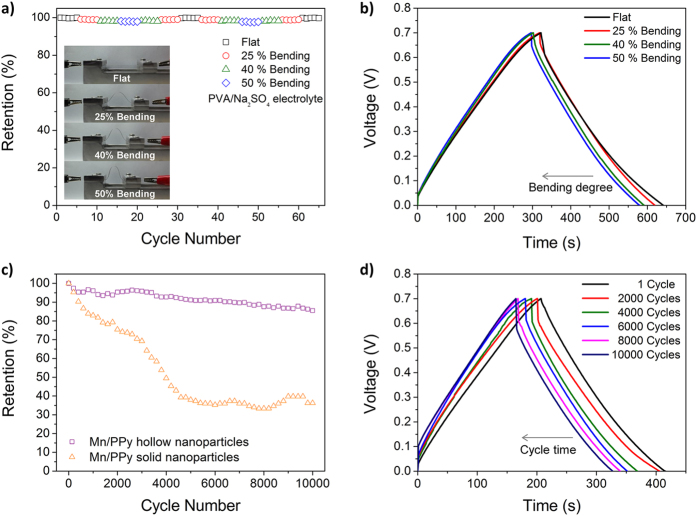
Flexible, all-solid-state cells. (a) Pictorial representation of an all-solid-state flexible electrochemical capacitor cell; (b) Typical galvanostatic charge/discharge curves of metal/PPy hollow nanoparticles measured at a current density of 0.1 A g^−1^; (c) Specific capacitances measured at different current densities; and (d) Calculated coulombic efficiencies.

**Figure 7:**
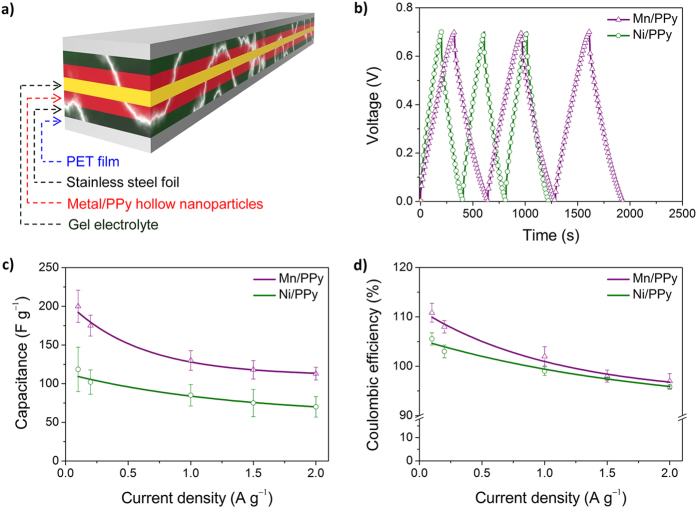
Flexible, all-solid-state cells based on Mn/PPy hollow nanoparticle electrodes. (**a**) Variation in the capacitance for different bend radii (the insets display the consequent changes in shape of the cell at each bend radius). (**b**) Galvanostatic charge/discharge curves recorded at a current density of 0.1 A g^−1^ under different bending conditions. (**c**) Plots of capacitance retention against cycle, in which solid nanoparticles were employed as a control. (**d**) Typical charge/discharge curves recorded over entire cycles.

Finally, an all-solid-state flexible electrochemical capacitor was prepared using the metal-decorated hollow nanoparticles as the electrode material. As illustrated in Fig. 6a, the capacitor cell wrapped with PET film, contained a polyvinyl alcohol (PVA)/Na_2_SO_4_ gel electrolyte as a separator and two stainless steel foils as the current collector. Figure 6b–d show the experimental data obtained through galvanostatic charge/discharge measurements. The Mn/PPy hollow nanoparticles were found to have longer discharging times without a drop in *IR*, as can be seen in Fig. 6b. The discharge capacitances of the Mn/PPy and Ni/PPy hollow nanoparticle electrodes were determined to be 113–200 F g^−1^ and 70–118 F g^−1^, respectively (Fig. 6c), and their coulombic efficiencies were excellent (>96%) (Fig. 6d). To evaluate the electrode performance as a flexible capacitor cell, a Mn/PPy hollow nanoparticle-based capacitor cell was chosen and tested under bending conditions (see Fig. 7a inset photos). The capacitor cell was found to show only 2% decrease in specific capacitance when it was bent up to 50%, as shown in Fig. 7a,b. The minor fluctuation in capacitance appeared to be reversible over the bending cycle. The cycling stability of the capacitor cell was then examined (Fig. 7c,d). We observed that the Mn/PPy hollow nanoparticles displayed an excellent capacitance retention of 86%, even following 10,000 charge/discharge cycles, which was 10% better than that obtained for the control, *i.e.*, the Mn/PPy solid nanoparticles (76%). In addition, the performance of metal-decorated electrode materials can be degraded by leaching of the metal ion during the charge/discharge cycling. Notably, PPy has a nitrogen atom in its repeating unit, which can chemically interact with metal cations^35^. The hollow nanoparticles could therefore act structurally as a nanocage to prevent the metal ion leaching from the inner space, thus leading to enhanced long-term cycling stability.

## Conclusions

PPy hollow nanoparticles with controlled diameters were successfully prepared through surfactant-templated chemical oxidation polymerization. The hollow nanoparticles had excellent potential for use as pseudocapacitive electrode materials with good redox activity. PPy was shown to act as a reducing agent for metal precursors, and was also found to be electrically conductive. Accordingly, the hollow nanoparticles were readily decorated with metal nanoclusters using chemical and electrochemical deposition methods. The resulting metal-decorated hollow nanoparticles served as high-capacitance electrode materials with good cycling stability in all-solid-state flexible capacitor cells, which was attributable to the structural benefits of the hollow nanoparticles. Various functional components can be introduced to the hollow nanoparticles, which might facilitate extended application of the conducting polymer hollow nanoparticles.

## Methods

### Materials

Pyrrole (98%), SDBS (technical grade), sulfuric acid (95–98%), sodium sulfate (>99%), nickel nitrate hexahydrate (99.999%), potassium permanganate (99%), 1-aminopyrene (97%) and PVA (Mw 90,000) were purchased from Aldrich. Ferric chloride (97%) was purchased from Merck. Poly(vinylidene) fluoride (PVDF) (KF1300 binder, Kureha) was used as a control binder, and 1-methyl-2-pyrrolidone (NMP) (Aldrich, 99.5%) was employed as a solvent to dissolve the binders. Distilled water was used for all experiments.

### Synthesis of PPy hollow nanoparticles

SDBS (40 wt%) was magnetically stirred in distilled water (40 mL) at room temperature. Pyrrole monomer (18.5 wt%, 14.9 mmol) was added dropwise to the surfactant solution, and a solution of ferric chloride (40.1 wt%) dissolved in distilled water (5 mL) was added to the surfactant/pyrrole solution. The chemical oxidative polymerization was allowed to proceed for 2 h at room temperature. The resulting product was washed with excess methyl alcohol and the resulting nanoparticle powder was dried in a vacuum oven at room temperature.

### Decoration of metal nanoclusters

While manganese species were chemically deposited on the hollow nanoparticles, nickel species were electrochemically deposited on the nanoparticles. For the chemical deposition of manganese on the nanocluster, PPy hollow nanoparticles (0.1 g) were mixed with a solution of KMnO_4_ (1 mL, 0.01 M) in water (10 mL). The resulting product was washed by suction filtration with excess water. Prior to electrodeposition, PPy hollow nanoparticles (2 g) were sonicated in a solution of nickel nitrate (10 mL, 0.01 M). Electrodeposition of nickel was carried out in a solution of H_2_SO_4_ (10^−4^ M) at −1.5 V for 15 min with a platinum counter electrode and Ag/AgCl reference electrode. The resulting hybrid nanoparticles were mixed with a solution of PVDF/NMP (12:1 *w/w*, 0.01 mL) and coated on a stainless steel plate, which was employed as a working electrode after drying in vacuum at 25 °C.

### Electrochemical measurements

CV and galvanostatic charge/discharge experiments were performed in a three-electrode cell containing a 1 M Na_2_SO_4_ solution as the electrolyte, and using a Pt auxiliary electrode and an Ag/AgCl reference electrode. The electrode material was dispersed in NMP (40 μL) containing PVDF (5 wt%) and was then coated onto stainless steel as a working electrode (95 wt%). Discharge specific capacitance was calculated using EIS. Nyquist plots were recorded in the frequency range of 100 MHz to 1 MHz. All electrochemical measurements were carried out using a Metrohm Autolab B.V. PGSTAT101 potentiostat/galvanostat.

### Flexible solid-state capacitor cells

Metal/PPy hollow nanoparticles were deposited on a stainless steel foil (area = 8 mm × 15 mm; thickness = 0.001 in) as a current collector. A polymer gel film was prepared using PVA (1 g), distilled water (20 mL), and a Na_2_SO_4_ solution (10 mL, 0.08 M), and the resulting film immersed in a solution of Na_2_SO_4_ (1 M). The resulting polymer gel electrolyte was inserted as a separator between the two metal/PPy hollow nanoparticle-coated stainless steel foils, and finally the stacked film/foils were encased using a polyethylene terephthalate coating.

### Characterization

For fluorescence probing, 1-aminopyrene was solubilized at 0.1 μM in water. Subsequently, SDBS and/or pyrrole were added to the solution. The morphology of the nanoparticles was observed using a JEOL EM-2000 EX II microscope. UV-Vis-NIR spectra were recorded using JASCO V-670. XPS was performed using a Thermo VG Scientific Multilab 2000 spectrometer with an Mg/Al twin-anode excitation source. The specimens were pelletized and then mounted on the standard sample studs by means of double-sided adhesive tape. Peak fitting of the collected spectra was conducted using VG Avantage software supplied by the manufacturer.

## Additional Information

**How to cite this article**: Ahn, K.-J. *et al.* Surfactant-Templated Synthesis of Polypyrrole Nanocages as Redox Mediators for Efficient Energy Storage. *Sci. Rep.* **5**, 14097; doi: 10.1038/srep14097 (2015).

## Supplementary Material

Supplementary Information
