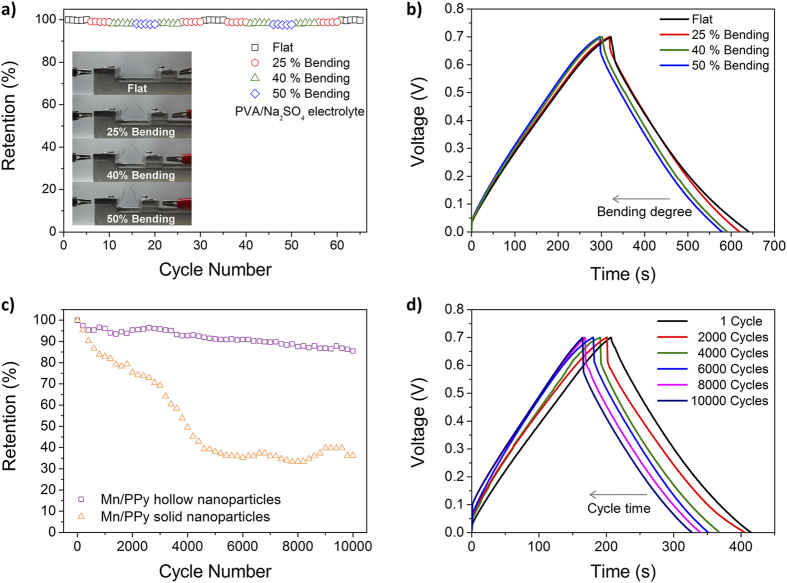# Corrigendum: Surfactant-Templated Synthesis of Polypyrrole Nanocages as Redox Mediators for Efficient Energy Storage

**DOI:** 10.1038/srep22501

**Published:** 2016-03-21

**Authors:** Ki-Jin Ahn, Younghee Lee, Hojin Choi, Min-Sik Kim, Kyungun Im, Seonmyeong Noh, Hyeonseok Yoon

Scientific Reports 5: Article number: 1409710.1038/srep14097; published online: 09162015; updated: 03212016

This Article contains an error in the order of the Figures. Figure 6 and Figure 7 were published as Figure 7 and Figure 6 respectively. The correct Figures 6 and 7 appear below as Figs 1 and 2 respectively. The Figure legends are correct.

## Figures and Tables

**Figure 1:**
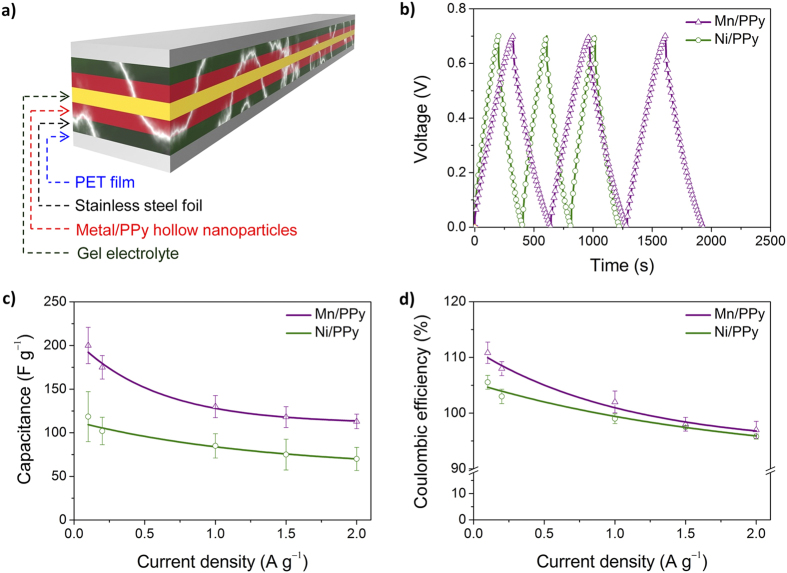


**Figure 2:**