# Supplementary material for: Surfactant-Templated Synthesis of Polypyrrole Nanocages as Redox Mediators for Efficient Energy Storage
Source: Sci Rep. 2015 Sep 16;5:14097. doi: 10.1038/srep14097 (PMC4571653; doi:10.1038/srep14097)
Supplement: Supplementary Information [file srep14097-s1.pdf]

Supporting Information

Surfactant-Templated Synthesis of Polypyrrole Nanocages as Redox Mediators for Efficient Energy Storage

Ki-Jin Ahn[b], Younghee Lee[b], Hojin Choi[b], Min-Sik Kim[b], Kyungun Im[b], Seonmyeong Noh[b], and Hyeonseok Yoon[a,b,*]

[a] Alan G. MacDiarmid Energy Research Institute, School of Polymer Science and Engineering, Chonnam National University, 77 Yongbong-ro, Buk-gu, Gwangju 500-757, South Korea. E-mail: hyoon@chonnam.ac.kr; Fax: +82-62-530-1779; Tel: +82-62-530-1778

[b] Department of Polymer Engineering, Graduate School, Chonnam National University, 77 Yongbong-ro, Buk-gu, Gwangju 500-757, South Korea.

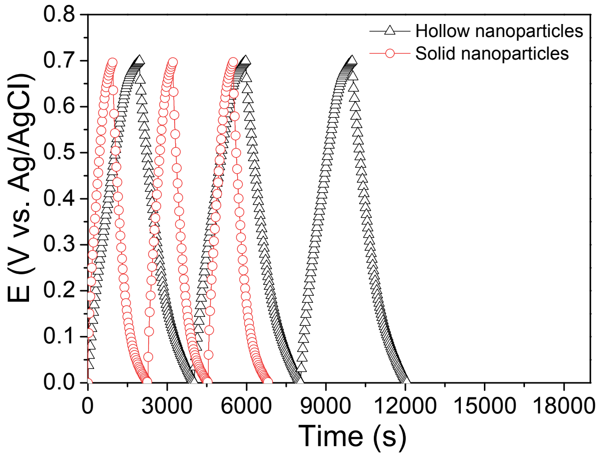

Figure S1. Galavanostatic charge/discharge curves of hollow vs. solid PPy nanoparticles.

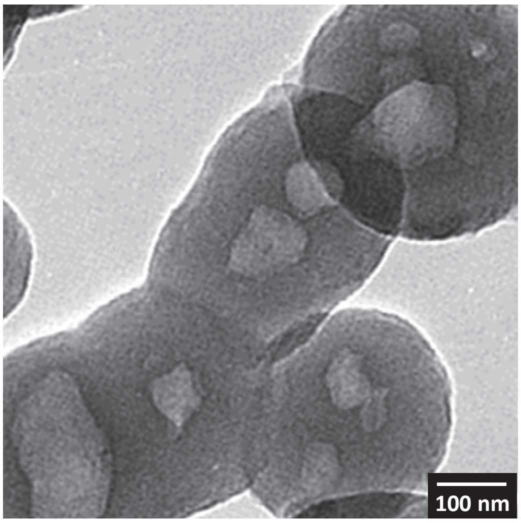

Figure S2. TEM image of PPy nanospheres containing multi-segmented interiors.
